# Attentional Disengagement Deficits Predict Brooding, but Not Reflection, Over a One-Year Period

**DOI:** 10.3389/fpsyg.2019.02282

**Published:** 2019-10-14

**Authors:** Eric S. Allard, Ilya Yaroslavsky

**Affiliations:** Department of Psychology, Cleveland State University, Cleveland, OH, United States

**Keywords:** attention, rumination, brooding, reflection, depression

## Abstract

A growing literature suggests that rumination is linked to attentional disengagement deficits in depression. This is particularly the case with brooding, a maladaptive form of rumination. However, research on the potential constructive association between attentional disengagement and self-reflection, a putative adaptive form of rumination, is sparse. Thus, the goal of the present study was to examine whether visual attentional disengagement deficits differentially predict dispositional brooding and self-reflection tendencies. Depressed participants (*n* = 17), those in remission from depression (*n* = 42), and their peers with no depression histories (*n* = 70) completed clinical interviews, the Ruminative Response Scale (RRS), and an eye-tracking task that measured attentional disengagement from pleasant (happy) and unpleasant (sad) facial images during a laboratory visit, and the RRS at 4 month intervals over a 1-year period. Results revealed that slow disengagement from sad faces, and rapid disengagement from happy faces, was specifically associated with brooding tendencies concurrently and across follow-up. Attentional disengagement was unrelated to self-reflection. The disengagement-brooding associations remained after controlling for depression status and anxiety disorder histories, suggesting that attentional control deficits may be a state-independent marker of brooding. Theoretical and clinical implications for these associations are discussed.

## Introduction

Failure to downregulate the intensity and duration of negative affective states, as appropriate to context, is a key vulnerability factor for depression and related psychopathology (Campbell-Sills and Barlow, 2007; Mennin et al., 2007; Joormann and Gotlib, 2010; Joormann and Quinn, 2014). Rumination, which reflects perseverative self-focused attention concerning the causes, meaning, and consequences of negative affect, has been shown to intensify and prolong negative affect and is a risk factor for depression (Nolen-Hoeksema, 2000; Spasojević and Alloy, 2001; Nolen-Hoeksema et al., 2008). Indeed, findings from laboratory studies show that experimentally induced ruminative states maintain dysphoria following negative mood induction (Nolen-Hoeksema and Morrow, 1993; Blagden and Craske, 1996), and meta-analytic reviews show robust associations between rumination and depression severity (Aldao et al., 2010), as well as depression status (Visted et al., 2018). Further, ruminative tendencies endure after the remission of depressive episodes (Visted et al., 2018) and prognosticate a risk for episode recurrence (Kovacs et al., 2009, 2016; Michalak et al., 2011).

Rumination is not a unitary construct and is comprised of self-focused attentional modes that are differentially linked to depression and negative affect (Treynor et al., 2003; Verhaeghen et al., 2005; Burwell and Shirk, 2007; Rude et al., 2007). For instance, brooding – or self-critical moody pondering – is considered maladaptive, as it is robustly associated with contemporaneous depression levels (Abela et al., 2002; Goodyer et al., 2003; Treynor et al., 2003; Watkins, 2004, 2009; Aldao et al., 2010; McLaughlin and Nolen-Hoeksema, 2011; Arnarson et al., 2016; Schäfer et al., 2017) and their worsening over time (Abela et al., 2002; Sarin et al., 2005; Roelofs et al., 2009; Calvete et al., 2015; Hudson et al., 2015; Gomez-Baya et al., 2017). Conversely, self-reflection – or the purposeful and non-judgmental appraisal of the positive or neutral content of distressing events – is believed to be an adaptive form of self-focused attention that facilitates problem solving (Treynor et al., 2003; Kross et al., 2005) and supports emotional well-being (Brans et al., 2013). Notably, the relationship between self-reflection and depression is less clear than that for brooding, with some studies finding no association between reflection and depression outcomes (Koval et al., 2012; Jose and Weir, 2013; Moore et al., 2013; Johnson et al., 2016; Tsypes and Gibb, 2016; Junkins and Haeffel, 2017; Artiran et al., 2019), others showing no distinction between its effects and those of brooding (Johnson and Whisman, 2013; Wilkinson et al., 2013; Padilla Paredes and Calvete Zumalde, 2015), and yet others observing the positive benefits of reflection on depression-relevant processes (Joormann et al., 2006; Burwell and Shirk, 2007; Arditte and Joormann, 2011). Thus, while brooding and reflection are forms of self-focused attention, the two differ as to the target of salient focus and perhaps relate to divergent affective outcomes.

### Impaired Attentional Control, Brooding, Reflection, and Depression

Some cognitive models of depression suggest that rumination (namely brooding) arises in part from attentional deficits or a reduced capacity to flexibly deploy and withdraw attention from internal and external sources generally concerning negative information when in a negative mood state (attentional disengagement; De Raedt and Koster, 2010; Whitmer and Gotlib, 2013). The “impaired disengagement hypothesis” in particular posits that attentional disengagement deficits have a synergistic relationship with brooding when confronted with a stressful event (Koster et al., 2011); such deficits result in a narrowed attentional focus that probalistically increase a brooding response that engenders further distress in lieu of a more effective strategy to manage distress (Koster et al., 2011). In a similar vein, the attentional scope model posits that dysphoric and low positive moods facilitate perseverative negative information processing concerning one’s emotional state and problems (Whitmer and Gotlib, 2013).

Growing empirical evidence links impaired attentional disengagement with brooding. For instance, previous studies have observed behavioral evidence of impaired attentional disengagement when individuals are processing both non-emotional (Davis and Nolen-Hoeksema, 2000; De Lissnyder et al., 2011) and emotional stimuli (Joormann, 2006; Joormann and Gotlib, 2008; De Lissnyder et al., 2012a), and such deficits are related to dispositional brooding (Koster et al., 2011). Furthermore, difficulty switching attention away from negative emotional (and toward neutral) affective targets is linked with trait brooding (De Lissnyder et al., 2012b). Additional evidence from an affective probe discrimination task shows that brooding is associated with slow attention disengagement from negative distractor words (Southworth et al., 2017). Similarly, dispositional tendencies to brood are associated with sustained visual attention on sad faces, which indirectly suggests attentional inflexibility (Owens and Gibb, 2017). Finally, using an eye tracking task that delineated attentional engagement from disengagement in response to emotional and neutral valenced facial pairs, greater habitual brooding was predictive of slower disengagement from negative faces, particularly when experiencing high levels of daily stress (Sanchez-Lopez et al., 2019). Thus, perseverative processing of depression-relevant stimuli (likely reflective of inflexible disengagement from negative affective elicitors) could be a key antecedent to brooding.

Though not fully articulated by either Koster’s or Whitmer’s model, a self-reflective response to negative self-relevant information may prove adaptive if the capacity to disengage attention from negative content when such information no longer poses a threat to well-being is intact. Empirical findings have associated self-reflection with enhanced attentional flexibility over self-relevant information (Daches et al., 2010), adaptive primary and secondary control strategies such as problem solving, cognitive restructuring, and acceptance (Burwell and Shirk, 2007), effective problem solving efforts (Haigh et al., 2018), and enhanced affective gains when problem-solving in daily life (Mori et al., 2015). As flexible attention is believed to support the effectiveness of adaptive coping and problem solving efforts (Ochsner and Gross, 2005; Teper and Inzlicht, 2013), there is indirect evidence for the role of attentional disengagement in the salubrious effects of self-reflection, which are well-aligned with the expected benefits of intact attentional disengagement that are proposed by the impaired disengagement hypothesis (Koster et al., 2011) and with a broadened attentional scope (Whitmer and Gotlib, 2013).

### Present Study

Though considerable efforts have been made to test the role of attentional processes on depression risk and brooding, most investigations employ cross-sectional designs with non-clinical samples. Results from these studies, while providing insight into depression risk, are also limited by potential range restriction in brooding levels that are higher among clinical samples and by their inability to disambiguate whether their findings concern the trait or state component of rumination (see Bagby et al., 2004). Further, little is known concerning the potentially adaptive link between intact attentional disengagement capacities and self-reflection. The aims of the present study were therefore two-fold: (1) to test whether attentional disengagement difficulties, indexed via an eye-tracking task measuring disengagement from depression-relevant stimuli (sad and happy faces; see Yaroslavsky et al., 2019), show differential contemporaneous associations with the brooding and self-reflection facets of depressive rumination in a mixed clinical sample of adults with various depression and anxiety disorder histories and (2) explore whether such relationships persist with the stable/invariant components of brooding and reflection across a 1-year period. Guided by conceptual work (Koster et al., 2011; Whitmer and Gotlib, 2013) and our prior findings that associate attentional disengagement deficits with depressive rumination (Yaroslavsky et al., 2019), we hypothesized that slow disengagement from sad faces, and rapid disengagement from happy faces, would positively predict contemporaneous and enduring brooding tendencies. Conversely, we hypothesized an inverse pattern of associations between the two attention disengagement indices and self-reflection. To contextualize our findings, we include the Ruminative Response Scale total score in our analyses from which the brooding and self-reflection indices arise, and test the clinical validity of the self-reflection index relative to contemporaneous depression symptoms and diagnostic status.

## Methods

### Participants

One hundred twenty-nine participants (88% male, M_*age*_ = 22.11, *SD* = 8.16) with normal or corrected-to-normal vision were recruited through online advertisements, referrals from outpatient treatment facilities, and from an undergraduate psychology student subject pool. Though we sought to recruit a balanced sample of participants with (*n* = 70) and without depression histories (*n* = 70), which would enable us to detect small-to-medium effect sizes within general linear models (Cohen’s *f*^2^ = 0.057), our recruitment efforts were limited by budgetary constraints. Racial background of participants was 67% Caucasian, 19% African American, 6% Latinx, 2% Middle Eastern, 2% multi-racial, and 4% who endorsed the South Asian, South East Asian or “other” category. Fifty-nine participants (46%) reported lifetime histories of a Depressive Disorder (*n* = 59 Major Depressive Disorder, *n* = 3 Dysthymic Disorder), of whom *n* = 17 were in the midst of a depressive episode (*n* = 15 Major Depression, *n* = 2 Dysthymic Disorder). Thirty (51%) of those with a lifetime history of Depressive Disorders also reported lifetime anxiety disorder histories (34% Social Anxiety Disorder, 6% Specific Phobia, 4% Obsessive Compulsive Disorder, 15% Panic Disorder, 33% Generalized Anxiety Disorder, 4% Post Traumatic Stress Disorder). Of the participants (*n* = 70) with no Depressive Disorder histories, *n* = 19 (27%) reported a lifetime history of anxiety disorders (16% Social Anxiety Disorder, 3% Specific Phobia, 2% Obsessive Compulsive Disorder, 6% Panic Disorder, 12% Generalized Anxiety Disorder, 2% Post Traumatic Stress Disorder), with the remaining *n* = 51 participants denying lifetime histories of psychiatric disorders.

### Interview and Self-Report Measures

#### Diagnostics Status

Diagnostics status was ascertained via the Structured Clinical Interview of DSM-IV Disorders (SCID-I; First et al., 1994) by advanced graduate students and one of the authors (IY). The SCID-I is a well-validated measure of psychiatric disorders that evidenced good inter-rater reliability in this study (SCID-I, Fleiss’ κs = 0.73–0.90). Diagnostic histories were determined during case consensus meetings following diagnostic consensus guidelines.

#### Depression Symptoms

Depression symptoms were measured via the self-rated Center for Epidemiological Depression Scale (CES-D; Radloff, 1977), a validated and reliable 20-item measure of symptoms during the prior 1 week period (α = 0.92 in this study).

#### Rumination

Rumination was measured via the Ruminative Response Scale (RRS), a 22-item survey of tendencies to brood and to reflect on one’s negative mood state (Treynor et al., 2003). The Brooding and Reflection subscales of the RRS are each comprised of 5 items and respectively, measure tendencies toward moody pondering and non-judgmental self-evaluation. The RRS and its subscales are well validated and had good internal consistency properties in this study across observations (RRS: αs = 0.93–0.96; Brooding: αs = 0.78–0.89; Reflection: αs = 0.70–0.83).

### Eye Tracking Measures

#### Task and Stimuli

The stimuli were face pairs that comprised emotional and neutral expressions. Faces were taken from the Karolinska Directed Emotional Faces (KDEF) database (Lundqvist et al., 1998) according to validation data from Sanchez et al. (2013). Based on a similar design from Sanchez et al. (2017), KDEF frontal view pictures that displayed discrete expressions of happiness, disgust, and sadness were used. A total of 24 happy, 24 sad, and 24 disgust face stimuli (12 men and 12 women for each emotion category, along with each actor’s neutral expression stimulus) were selected for the eye tracking (ET) task.

**FIGURE 1:**
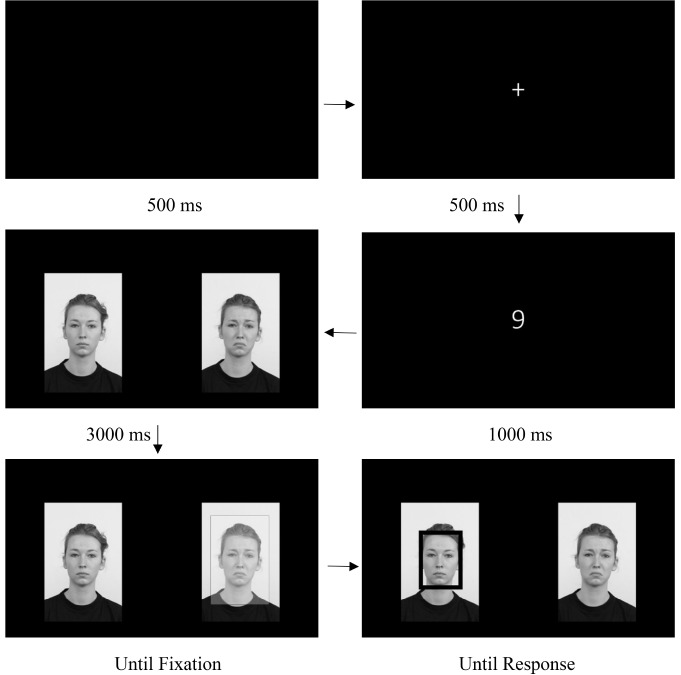
Schematic outline of the ET procedure. The arrows denote the temporal order of the stimulus presentation. Note that for one-third of the trials, the “Until Fixation” and “Until Response” stimuli were not presented. Figure reproduced with permission from Yaroslavsky et al. (2019).

The ET comprised 72 trials (24 happy-neutral, 24 sad-neutral, and 24 disgust-neutral pairs). Face stimuli were displayed on a 48 cm (width) × 27 cm (height) widescreen computer monitor. Each image was 12 cm (width) × 18 cm (height). Faces were centered on the screen, at a distance of 25 cm from the center of each image. Participants were approximately 60 cm from the center of the computer monitor. This resulted in a visual angle of approximately 11.8° between each image’s center and the center of the screen. The experimental design was similar to one used in Sanchez et al. (2013). Each trial started with a black screen for 500 ms, followed by a central fixation cross for another 500 ms. A single, random digit (i.e., 1–9) replaced the fixation cross and remained for 1,000 ms; participants were instructed to say this number aloud so as to ensure their attention was oriented to the center of the screen prior to the face pair presentation. Immediately after digit offset, the faces appeared on the screen for a 3,000-ms “free viewing” period (i.e., participants were instructed to “view the images naturally as if at home watching television”), after which a new trial began one-third of the time. Another third of the trials assessed attentional engagement with the emotional face in the pair: participants had to disengage gaze from the neutral face in order to engage gaze with the emotional face in the pair. The final third of the trials assessed attentional disengagement from the emotional face in the pair: participants had to disengage gaze from the emotional face in order to engage gaze with the neutral face in the pair. For the emotional engagement trials, after “free viewing,” participants’ fixation on the neutral face (for 100 ms) triggered a rectangular or oval frame to appear around the opposite emotional face in the pair, and participants indicated the shape of the frame by pressing one of two keys on a keyboard corresponding to a “rectangle” or “oval.” For the disengagement trials, the opposite occurred. In other words, a 100-ms fixation on the emotional face triggered a rectangle or oval frame to surround the neutral face, and disengagement was determined by the amount of time needed to shift gaze from the emotional face toward the neutral face. Participants completed two practice trials to ensure their understanding of task instructions. All three trial conditions were randomly presented, and both types of frames and valenced faces were equal in their presentation and whether or not they appeared in the left and right positions during the engagement and disengagement conditions. Emotional and neutral faces were equally presented on the left and right side of the screen across trials. A schematic example of a trial sequence can be found in Figure 1. The disengagement indices used in this study reflect the average delay (in ms) to first fixation on the target face (i.e., the face surrounded by a rectangle or oval), and were computed via Huber M estimation to down-weigh the influence of outliers (see Wilcox, 2017) comprising 1.5 and 1.7% of sad and happy face disengagement trials, respectively. Both disengagement indices displayed adequate internal consistency (Sad Disengagement α = 0.68, Happy Disengagement α = 0.70).

#### Eye Tracking Apparatus

Eye movements were recorded using the RED-m (Sensomotoric Instruments, SMI; Berlin, Germany) and Tobii X3-120 (Tobii Technology, Inc.) 120 Hz eye-tracking devices in the authors’ labs, respectively. Both stimulus presentation and eye movement recording was conducted using SMI Experiment Center and E-prime v. 2 software. Visual fixations were defined as gaze resting within 0.5–1.0° visual angle for at least 100 ms (Manor and Gordon, 2003) within pre-determined areas of interest (AOIs). AOIs comprised the entirety of the facial stimuli for both the free-viewing and engage-disengage tasks.

### Procedure

Participants completed clinical evaluations, survey measures, an hour-long protocol that assessed physiological and psychological reactions to various stimuli/tasks, and the ET task during a four-hour laboratory session. They were then contacted 4-, 8-, and 12-months following their laboratory visit and completed a set of survey measures online that included the RRS. Time between follow-up assessments was approximately 4 months (*Ms* = 3.66–4.37, *SDs* = 0.88–1.01), with *n* = 102 (79%) participants providing data on 2 occasions, *n* = 85 (66%) on 3 occasions, and *n* = 72 (56%) completing all assessments.

### Statistical Analyses

Descriptive and inferential analyses were conducted using SAS version 9.4 software (SAS Institute Inc., 2013). Pearson Chi-Square and Analyses of Variance (ANOVAs) that employed Tukey’s HSD to correct for multiple comparisons were used to characterize the sample with respect to the three diagnostic groups. Bivariate correlations were calculated between clinical characteristics ascertained during the laboratory visit and contemporaneously measured Brooding and Reflection subscales of the RRS to test the clinical validity of the two subscales, as were regression models to test contemporaneous effects of attention indices on the Brooding and Reflection subscales, while covarying effects of demographic and clinical characteristics. Mixed effects models were employed to test associations between attention indices and time-invariant components of the RRS and its two subscales in order to accommodate the multi-level structure of the longitudinal data and were fit using Restricted Maximum Likelihood and empirical standard errors to accommodate moderate heteroscedasticity (Liang and Zeger, 1986). In these models, Level 1 dependent variables were the RRS and its subscales, which displayed high-to-moderate stability across follow-up assessments (ICC_*RRS*_ = 0.73, ICC_*Brood*_ = 0.67, ICC_*Reflect*_ = 0.55, *ps* < 0.001), and the two attention disengagement indices served as the Level 2 predictors of interest (*Sad Dis*. and *Hap. Dis*). Level 2 covariates included age and sex, as were depression status (*C. Dep* and *P. Dep*) and anxiety disorder histories (*Anx*) in light of prior findings that associate attentional disengagement deficits with both disorder classes (Ellenbogen and Schwartzman, 2009; Reinholdt-Dunne et al., 2013). Continuous predictors and covariates were grand mean centered, while dichotomous predictors retained their original metric. The equations below reflect all fixed (γ) and random (u) effects, with ${\hat{Y}}_{i\,j}$ reflecting the predicted value on the RRS, Brooding, or Reflection subscales on observation *i* for participant *j.* Variance components along with first- and second-order autoregressive structures were imposed on the random effects and residual variance-covariance matrices, respectively, to accommodate the single random effect (i.e., random intercept) and potential residual auto-correlations. However, the residual variance-covariance matrix was excluded from the final models to avoid model over-fitting, given that the inclusion of auto-regressive parameters did not significantly improve model fit (Δ−2LL = 0.4–2.20, *ps* = 0.14–0.53; Snijder and Bosker, 2012).

Level 1 equation

$${\hat{Y}}_{i\,j}=\beta_{0\,j}$$

Level 2 equations

$$\beta_{0\,j}=\gamma_{00}+\gamma_{01}\,A\,g\,e+\gamma_{02}\,S\,e\,x+\gamma_{03}\,A\,n\,x+\gamma_{04}\,C.D\,e\,p+\gamma_{05}\,P.D\,e\,p+\gamma_{06}\,S\,a\,d\,D\,i\,s.+\gamma_{07}\,H\,a\,p.D\,i\,s.+u_{0\,j}$$

## Results

### Preliminary Analyses

Group differences in demographic, clinical, and attentional characteristics of depressed, remitted, and control participants are presented in Table 1. Depressed participants were significantly older than their remitted and control peers, but otherwise the three groups did not differ in their demographic characteristics. Groups evidenced a rank order in depression symptoms, with those in the midst of a depressive episode reporting the highest levels that were followed by remitted and control participants. Those in the remitted group evidenced more abundant histories of anxiety disorders than control participants, while depressed participants did not significantly differ from the other two groups. The three groups showed similar patterns of attentional disengagement from positive and negative valenced faces.

**TABLE 1:** Demographic, clinical, and attentional characteristics among control, remitted, and depressed participants.

**Variable**	**Control (*n* = 70)**	**Remitted (*n* = 42)**	**Depressed (*n* = 17)**	**Test Statistic**
Age	19.76 (3.00)^a^	24.17 (9.74)^a^	26.65 (13.91)^b^	*F*(2, 126) = 7.58, *p* < 0.001, η^2^ = 0.11
Sex (Female)	67%	76%	52%	χ^2^ (2) = 3.10, *p* = 0.21, *V* = 0.16
Race^†^				χ^2^ (4) = 4.58, *p* = 0.31, *V* = 0.13
African A	14%	22%	29%	
Caucasian	72%	64%	71%	
Other	14%	14%	0%	
Anxiety Hx	27%^a^	55%^b^	41%^a,b^	χ^2^ (2) = 8.59, *p* = 0.01, *V* = 0.26
CES-D	9.79 (7.64)^a^	17.07 (9.20)^b^	29.82 (8.71)^c^	*F*(2, 126) = 7.58, *p* < 0.001, η^2^ = 0.40
Sad Disengagement	246.99 (65.16)	271.47 (94.74)	251.55 (76.24)	*F*(2, 126) = 1.34, *p* = 0.27, η^2^ = 0.02
Happy Disengagement	246.79 (54.93)	259.32 (71.86)	241.15 (60.04)	*F*(2, 126) = 0.75, *p* = 0.47, η^2^ = 0.01

*Group values represent means and standard deviations or percent. Anxiety Hx = lifetime history of an anxiety disorder diagnosis; CES-D = Center for Epidemiologic Studies Depression Scale; Sad Disengage = time to first fixation in milliseconds from a sad-valenced face to a neutral-valenced face; Happy Disengage = time to first fixation in milliseconds from a happy-valenced face to a neutral-valenced face. *Post hoc* contrasts are corrected for multiple comparisons using Tukey’s HSD, and values sharing a letter in their superscript do not significantly differ (*p* > 0.05). †Racial categories were aggregated, and the Fisher Exact test was employed to accommodate sparse cell frequencies.*

Consistent with the extant literature, scores on the Brooding subscales positively correlated with depression symptoms, *r*(127) = 0.57, *p* < 0.001, and anxiety disorder histories at a non-significant trend level, *r*(127) = 0.17, *p* = 0.06. Notably, the Reflection subscale scores also positively correlated with depression levels, *r*(127) = 0.38, *p* < 0.001, anxiety disorder histories, *r*(127) = 0.18, *p* = 0.04, and those of the Brooding subscale, *r*(127) = 0.64, *p* < 0.001. Therefore, contrary to expectation, the Reflection subscale did not evidence clinical validity, given its positive associations with indices of psychopathology nor distinction at the construct level from the Brooding subscale.

### Do Attentional Disengagement Deficits Predict Trait Rumination, Brooding, and Reflection?

Results from regression and mixed effects models are presented in Tables 2, 3. As hypothesized, delayed disengagement from sad faces robustly predicted elevated tendencies to ruminate (β*_*RRS*_* = 0.18, γ*_*RRS*_* = 0.036, *ps* = 0.013 − 0.03) and to brood (β*_*Brood*_* = 0.19, γ*_*Brood*_* = 0.01, *ps* = 0.02 − 0.03) across the laboratory and follow-up assessments, as did rapid disengagement from happy faces (β*_*Brood*_* = −0.20, γ*_*RRS*_* = −0.032, *ps* = 0.005 − 0.03; β*_*Brood*_* = −0.23, γ*_*Brood*_* = −0.033, *ps* = 0.007−0.05). Importantly, these effects were independent of depression status and anxiety disorder histories, which significantly predicted both outcomes and suggest that the effects of attentional disengagement deficits are state-independent and enduring. Of note, and in contrast to expectation, neither disengagement from sad nor happy faces predicted trait reflection levels across the follow-up period, though akin to brooding, fast disengagement from happy faces predicted a contemporaneous tendency toward reflection at a trend level. Indeed, of the covariates, only depressive disorder histories evidenced a significant positive relationship with reflection levels, γ = 1.11, *t* (121) = 2.04, *p* = 0.04.

**TABLE 2:** Demographic characteristics, psychiatric history, and attention disengagement index predictions of contemporaneous ruminative response scale total and the brooding and reflection subscales scores.

	**RRS**			**Brooding**			**Reflection**		
**Variable**	**B**	**SE**	**β**	**B**	**SE**	**β**	**B**	**SE**	**β**
Age	−0.37^∗^	0.16	–0.21	–0.14^∗∗^	0.04	–0.28	–0.00	0.09	–0.00
Sex	1.21	2.38	0.04	–0.01	0.67	–0.00	0.62	0.57	0.08
Current dep. D/O	14.42^∗∗∗^	3.36	0.34	3.61^∗∗∗^	0.93	0.31	2.09^†^	1.10	0.21
Past dep. D/O	5.54^∗^	2.55	0.19	1.63^∗^	0.69	0.20	0.54	0.68	0.08
Anx. D/O Hx	4.13	2.48	0.14	0.82	0.70	0.10	1.15^†^	0.62	0.16
Sad disengage	0.033^∗^	0.013	0.18	0.010^∗^	0.004	0.19	0.004	0.004	0.09
Happy disengage	–0.047^∗∗^	0.016	–0.20	–0.015^∗∗^	0.005	–0.23	−0.009^†^	0.005	–0.17
*R*^2^		0.25			0.26			0.12	

*RRS = Ruminative Response Scale total score, Dep. D/O = depressive disorder (major depression, dysthymic disorder, or depressive disorder NOS), Anx. D/O Hx = history of anxiety disorder (generalized anxiety disorder, social anxiety disorder, panic disorder, specific phobia, and post-traumatic stress disorder); Sad Disengage = time to first fixation in milliseconds from a sad-valenced face to a neutral-valenced face; Happy Disengage = time to first fixation in milliseconds from a happy-valenced face to a neutral-valenced face. ^∗∗∗^*p* ≤ 0.001, ^∗∗^*p* ≤ 0.01, ^∗^*p* < 0.05, and ^†^*p* < 0.07.*

**TABLE 3:** Demographic characteristic, psychiatric history, and attention disengagement index predictions of invariant ruminative response scale total and the brooding and reflection subscales scores.

**Variable**	**RRS γ (SE)**	**Brooding γ (SE)**	**Reflection γ (SE)**
Age	−0.32^∗^(0.14)	−0.09^∗∗^(0.03)	−0.04(0.04)
Sex	−0.54(2.04)	0.10 (0.55)	−0.34(0.51)
Current dep. D/O	12.88^***^(3.26)	3.56^***^(0.99)	1.11 (0.95)
Past dep. D/O	6.90^∗∗^(2.34)	1.55^∗∗^(0.59)	1.21^∗^(0.59)
Anx. D/O Hx	4.85^∗^(2.24)	1.25^∗^(0.58)	0.78 (0.55)
Sad disengage	0.036^∗^(0.016)	0.01^∗^(0.004)	0.005 (0.004)
Happy disengage	−0.033^∗^(0.016)	−0.011^∗∗^(0.004)	−0.002(0.005)
**Random Effects**			
Intercept	119.45^***^(18.74)	7.56^***^(1.26)	6.84^***^(1.19)
Residual	60.57^***^(5.43)	5.07^***^(0.45)	5.98^***^(0.53)
Pseudo *R*^2a^	0.26	0.27	0.05

*RRS = Ruminative Response Scale total score, Dep. D/O = depressive disorder (major depression, dysthymic disorder, or depressive disorder NOS), Anx. D/O Hx = history of anxiety disorder (generalized anxiety disorder, social anxiety disorder, panic disorder, specific phobia, and post-traumatic stress disorder); Sad Disengage = time to first fixation in milliseconds from a sad-valenced face to a neutral-valenced face; Happy Disengage = time to first fixation in milliseconds from a happy-valenced face to a neutral-valenced face. ^*a*^Pseudo *R*^2^ approximates variance explained in the Random Intercept component by model predictors and covariates relative to the empty model. ^∗∗∗^*p* ≤ 0.001, ^∗∗^*p* ≤ 0.01, and ^∗^*p* < 0.05.*

## Discussion

Conceptual works and growing empirical evidence suggests that rumination, a well-known risk factor for depressive disorders (Nolen-Hoeksema et al., 2008), is associated with attentional disengagement deficits (Koster et al., 2011; Sanchez-Lopez et al., 2019; Yaroslavsky et al., 2019). However, not all ruminative thinking takes a depressogenic form (e.g., Brooding), as non-judgmental self-reflection has been linked in some studies to emotional well-being and effective problem solving (Kross et al., 2005; Brans et al., 2013). As the preponderance of studies on the relationship between attentional disengagement deficits and rumination focus on the maladaptive forms of self-focused attention, the relationship between attentional flexibility and self-reflection is not clear. We sought to address this gap in the literature by testing whether slow visual attentional disengagement from unpleasant (sad faces) and rapid disengagement from pleasant (happy faces) stimuli differentially predict the adaptive and maladaptive forms of self-focused attention. To wit, we examined associations between attentional disengagement and the Brooding and Reflection facets of the Ruminative Response Scale, a common measure of depressive rumination, that were contemporaneously measured with attention disengagement and across 12 months in a sample of adults with various depressive and anxiety disorder histories.

As hypothesized, slow attentional disengagement from sad faces was significantly predictive of ruminative tendencies, particularly brooding, during the initial laboratory assessment and over the course of the 1-year follow-up period, as was rapid disengagement from happy faces. These results are in line with prior work observing that slower disengagement from depression-relevant information is associated with greater levels of habitual brooding (Sanchez-Lopez et al., 2019). Additionally, the present results extend our prior findings as to the role of rapid disengagement from pleasant affective stimuli being linked with rumination (Yaroslavsky et al., 2019). This further evinces the role of attentional disengagement issues – in relation to pleasant and unpleasant affective elicitors – on the perseverative, negative self-relevant thoughts that are a hallmark of depressive symptomology (Koster et al., 2011). Importantly, the predictive associations between attentional disengagement and brooding were independent of depression status and anxiety disorder histories. This suggests that attentional disengagement problems may be a state-independent, and perhaps transdiagnostic, prognosticator of maladaptive self-focused attention.

Contrary to our hypotheses, no attentional disengagement indices were significantly predictive of self-reflection tendencies during the laboratory assessment or at follow-up. Thus, while flexible attentional disengagement has been shown to be adaptive in a variety of contexts (Ochsner and Gross, 2005; Teper and Inzlicht, 2013), the present study did not observe the salutary effects of flexible attentional disengagement on self-reflection. This was further evidenced by the lack of a significant correlation between the RRS Reflection subscale and depression levels in the present sample. Our results add to the mixed evidence for identifying the predictive relationships between self-reflection and depression-related outcomes (see Arditte and Joormann, 2011; Johnson and Whisman, 2013: Padilla Paredes and Calvete Zumalde, 2015; Johnson et al., 2016; Artiran et al., 2019).

The potential trait-based role of positive and negative attentional disengagement in predicting brooding – rather than self-reflection – is a novel contribution that could inform theory and practice regarding cognitive models of depression. Recent research has observed associations between training paradigms for treating attentional disengagement problems in response to negative affective elicitors (e.g., Attentional Bias Modification), observing that attentional training can work to alleviate prospective depression symptoms (Beevers et al., 2015; Yang et al., 2015) and episodic recurrence (Browning et al., 2012). One lingering question with such training paradigms is the mechanism of action linking attentional modification and depressive outcomes. It is plausible that improvements to attentional disengagement processes are most influential by acting upon negative perseverative brooding styles. Future work is needed to better determine how improving attentional disengagement capacities impacts depressive outcomes through influences on rumination processes.

## Limitations and Future Directions

Though this study has several strengths in the form of a well-characterized clinical sample, the use of a novel eye-tracking paradigm, and a longitudinal design, it is not without limitations. First, the sample was primarily comprised of Caucasian women, thereby limiting generalizability of our findings. Second, though attrition is a common problem in longitudinal studies, the reduction of our sample size limited the precision and power of our statistical analyses. Finally, though trait-like in its nature, ruminative thinking has been shown to fluctuate over time (Bagby et al., 2004). Due to attrition, we were unable to fit statistical models that could test the concomitant associations between attentional control indices and time-varying and invariant components of self-referential thinking. Future studies that recruit and retain a large and diverse clinical sample would do much to elucidate whether and in what way attentional disengagement differentially predicts the purported adaptive and maladaptive forms of rumination.

## Conclusion

The present findings further highlight associations, based on cognitive models of depression, between attentional disengagement difficulties and rumination, with a key link emerging for brooding relative to self-reflection. The fact that these associations were independent of depression status and anxiety disorder histories suggest that such attentional disengagement deficits may be a trait-based marker of problematic affect that undermines attempts at well-being for individuals experiencing current episode depression, remitted depression, and even among those without a prior depression history. Thus, interventions that target the insidious link between attentional disengagement difficulties and brooding could be key for reducing depression symptoms, enhancing relapse prevention, and protecting against first episode occurrence.

## Data Availability Statement

Data for independent replication of our findings are available from the corresponding authors upon request.

## Ethics Statement

The studies involving human participants were reviewed and approved by the Cleveland State University Institutional Review Board. The participants provided their written informed consent to participate in this study.

## Author Contributions

EA and IY contributed equally to the design and execution of the study protocol, as well as all aspects of manuscript preparation.

## Conflict of Interest

The authors declare that the research was conducted in the absence of any commercial or financial relationships that could be construed as a potential conflict of interest.
